# Recent Advances in the Modulation of Cholinergic Signaling

**DOI:** 10.3390/molecules27185971

**Published:** 2022-09-14

**Authors:** Clelia Dallanoce

**Affiliations:** Department of Pharmaceutical Sciences, Medicinal Chemistry Section “Pietro Pratesi”, University of Milan, Via L. Mangiagalli 25, 20133 Milan, Italy; clelia.dallanoce@unimi.it

In recent years, an impressive number of research studies have been conducted to improve the understanding of the structure and function of the cholinergic system, and significant progress has also been made in elucidating the roles of neuronal and non-neuronal acetylcholine (ACh) in the pathogenesis and treatment of human disease. Indeed, this research area is still very much developing, and this Special Issue explores new insights into cholinergic-signaling functions, their roles in protecting the body from disease, and how the structure of cholinergic modulators can be manipulated in view of therapeutic applications. It includes twelve current research papers and four reviews in this field.

Five research articles deal with the anticholinesterase activity of different compounds [1, 2, 3, 4, 5].

Makhaeva et al. [1] developed bis-amiridine derivatives joined by two different spacers, bis-*N*-acyl-alkylene and bis-*N*-thiourea-alkylene. The new compounds exhibited high inhibitory activity against both acetylcholinesterase (AChE) and butyrylcholinesterase (BChE) with a degree of selectivity towards the latter enzyme. In particular, bis-amiridines with thiourea-containing spacers showed a mild inhibition of Aβ_42_ self-aggregation, revealed high antioxidant activity, and exerted other potentially disease-modifying effects, thus displaying the properties of multitarget drug candidates for Alzheimer’s Disease (AD) therapy.

Krátký et al. [2] evaluated the inhibitory potency towards AChE and BChE of a series of hydrazones of 4-(trifluoromethyl)benzohydrazide. Most of the compounds were stronger inhibitors of AChE and only two derivatives characterized by the presence of 2-chlorobenzylidene and 2-(trifluoromethyl)benzylidene substituents were found to be more potent inhibitors of BChE than AChE. Following an in silico prediction of the blood–brain barrier’s (BBB) permeability with respect to reaching the target site, as well as the prediction of gastrointestinal (GI) absorption, the synthesized hydrazide–hydrazones showed generally good properties for both CNS delivery and GI absorption.

Suwanhom et al. [3] focused on the synthesis and anticholinesterase activity evaluation of quinoxaline derivatives. The new compounds showed potent inhibitory activity against AChE and only some of them against BChE. They did not exhibit significant toxicity towards the SH-SY5Y neuronal cells and were predicted to be able to permeate the BBB and exhibit good GI absorption. Unlike the known AChE inhibitors such as Tacrine and Galanthamine, the quinoxaline derivatives bind to the peripheral anionic site (PAS) of human AChE rather than the catalytic anionic site (CAS) and represent a promising lead scaffold for the further development of novel potential acetylcholinesterase inhibitors.

Two potent AChE inhibitors (IC_50_ 40–50 nM) characterized by the 3-nitro-6-amino-imidazo[1,2-*b*]pyridazine scaffold with a piperidine and 1-phenylpiperazine substituents were investigated by Sharma et al. [4] and resulted in multifunctional lead molecules that, aside from AChE inhibition, exhibit antiproliferative, antimigratory, and anti-inflammatory effects at higher doses.

Since the Lycopodiaceae alkaloids are among the potent acetylcholinesterase (AChE) inhibitors, Dymek et al. [5], for the first time, reported the isolation of pre-purified Lycopodiaceae alkaloid fractions combined with bioactivity testing and cytotoxicity evaluation of the extracts. Three plant species belonging to the Lycopodiaceae family were employed. Optimized pressurized liquid extraction and innovative gradient vacuum liquid chromatography, used for pre-purification, enabled the researchers to collect more than 100 fractions, which were analyzed via HPLC/ESI-QTOF-MS and in turn enabled the detection of more than 50 alkaloids. TLC bioautography assays proved that the three analyzed species are rich sources of AChE inhibitors, and *Huperzia selago* showed the highest anti-AChE activity. In addition, the extracts did not exhibit cytotoxicity towards skin fibroblasts. The entire process was carried out with a modern device, thereby allowing the researchers to reduce the overall time of the analysis and the consumption of the expensive enzyme.

Three research articles focus on studies involving the α7 nicotinic acetylcholine receptor (nAChR) signaling [6, 7, 8]. Salazar Intriago et al. [6] established that the selective activation of the α7 receptor may be relevant for promoting the microenvironment favorable for improving peripheral nerve regeneration. The authors observed that the expression of the α7 subtype receptor is significantly enhanced in Schwann cells of the rat sciatic nerve after a peripheral dissection or in the presence of the proinflammatory neuropeptide Bradykinin. They demonstrated that the selective activation of α7 nAChRs with the partial agonist ICH3 induces a decreased expression of the transcriptional factor c-Jun and upregulated activity of metalloproteinases, which are essential during the regenerative processes.

Navarro et al. [7] investigated how α7 nAChR stimulation modulates microglial phenotypes in an LPS-induced neuroinflammation model in adult and aged mice. The study showed that the activation of microglial α7 nAChR—through a co-administration of the α7 agonist PNU282987 with LPS—reduces LPS-induced behavioral sickness and inflammatory markers in adult but not in aged animals. Further investigations would be needed to define if α7 nAChR signaling is an effective target for controlling neuroinflammation in age-related diseases.

Duarte et al. [8] evaluated the rat α7 nAChR modulation via seven antidepressants (norfluoxetine, mirtazapine, imipramine, bupropion, fluoxetine, venlafaxine, and escitalopram) with different structures and pharmacological profiles. The investigation was performed by employing electrophysiological assays and molecular docking and dynamics simulations. The α7 nAChR was found to be negatively modulated by all the tested antidepressants, which similarly inhibit the ion current elicited by choline and interact within the α7 ion channel with slight differences in binding-free energy. These findings promote a better understanding of the interactions between antidepressants and α7 nAChRs and suggest that putative therapeutic or adverse effects of these drugs are attributed to their interaction with the α7 receptor.

The next article is an interesting investigation on the determinants for α4β2 over α3β4 nAChRs subtype selectivity. Bavo et al. [9] analyzed more than forty α4β2 ligands, which are pyrrolidine analogues of the α4β2 agonist A-84543. These compounds were docked into the structures of the human α4β2 and α3β4 nicotinic receptors, which were recently determined by cryo-electron microscopy. The authors evidenced that the key distinctive features for the α4β2 versus α3β4 selectivity are the stabilization of the ligand’s aromatic ring by the non-conserved β2-Phe119 residue and direct or water-mediated interaction with hydrophilic residues of the β minus side, which is influenced by the extensibility of the portion containing the aromatic ring and its decoration.

Two original papers present the involvement of cholinergic signaling in different pathologies. Linares et al. [10] studied the participation of ACh in the development of polycystic ovary syndrome (PCOS) in rats. PCOS was induced by an injection of estradiol valerate, and the rats were then treated with the nonselective cholinergic blocker atropine. The cholinergic system appeared to modulate the secretion of the steroid hormones—progesterone and testosterone—in a stimulatory way, while its participation in the regulation of the mechanisms that lead to follicle rupture seemed to depend on the availability of muscarinic receptor subtypes.

An investigation studying the gastrointestinal side effects of Donepezil, a potent reversible cholinesterase inhibitor used as the preferred treatment for Alzheimer’s disease, was performed by Bures et al. [11]. The pharmacokinetic parameters of Donepezil were evaluated in experimental pigs with and without a small intestinal injury induced by dextran sodium sulfate (DSS). The results indicated that both small intestinal injuries and a prolonged small transit time give rise to higher plasma concentrations of Donepezil, which also cause an unfavorable effect on porcine gastric myoelectric activity. This suggests that it would be necessary to reduce the doses of this drug in humans with gastrointestinal disease.

A further contribution by Hansen et al. [12] focuses on the pharmacological investigation of the DEG-3/DES-2 nAChR subtype. Unlike other nAChRs associated with worms’ motility, including subunits from the nematode-specific DEG-3 group, the DEG-3/DES-2 channel is involved in nociception and possibly in chemotaxis. The activity of the DEG-3/DES-2 channel from the parasitic nematode of ruminants, *Haemonchus contortus*, is modulated by the oral anthelmintic drug, monepantel, and its sulfone metabolite. Conversely, the authors documented that DEG-3/DES-2 from the free-living model nematode *Caenorhabditis elegans*—functionally expressed in *Xenopus laevis* oocytes and investigated using two-electrode voltage-clamp electrophysiology—was insensitive to monepantel. Therefore, pharmacological differences between non-parasitic and parasitic nematode species were evidenced, hinting that the nAChR subtypes of the DEG-3 group in certain parasites may be targets for the development of natural nematocidal compounds.

Besides the research papers, four interesting review articles [13,14,15,16] have been published in this Special Issue. Two of them deal with neurological disorders due to impaired ACh-related functions [13,14]. The first was written by Chen et al. [13] and summarizes the role of cholinergic signaling in Alzheimer’s Disease (AD). Although the pathogenesis of AD is complex and remains unclear, AD patients often manifest a deficiency in ACh and damage to cholinergic signal transduction, which is associated with cognitive decline and memory impairment. The current knowledge about other pathophysiological features—such as neuroinflammation, metabolic stress, and cerebrovascular and endothelial dysfunctions—is also presented. Additionally, the authors illustrated the most promising drugs and treatments of AD and underlined that acetylcholinesterase inhibitors (AChEIs) are the only drugs currently approved for the therapy of AD. The second review by Wang et al. [14] discusses the involvement of cholinergic signaling in epilepsy, which is mainly caused by the imbalance of excitation and inhibition. Both muscarinic and neuronal nicotinic AChRs can influence the intrinsic excitability of neurons and synaptic transmission. The results from several studies are reported, highlighting that cholinergic dysfunction is closely correlated with epilepsy at molecular, cellular, and circuit levels. These data suggest that circuit-level therapy targeting cholinergic neurons may be a promising option for handling epileptic patients.

A third review article, written by Nosaka et al. [15], focuses on the theory of striatal cholinergic interneuron signaling, for which numerous varicosities on the axon produce an extra-synaptic volume-transmitted, rather than mediated, rapid point-to-point synaptic transmission. The authors integrated the measurements available in the literature by applying a mathematical model, and their calculations, together with recent data from genetically encoded sensors, showed that ACh diffusion is both short-range and short-lived. Thus, these data suggest that ACh signaling by the cholinergic interneurons of the striatum should be a combination of local, transient peaks of ACh concentration adjacent to the release sites mediating the time-resolved transmission of spike timing to the postsynaptic cell, with a more distant pooling of ACh to create an ambient level.

Finally, this Special Issue includes a review article by Bekbossynova et al. [16] devoted to toxins targeting nAChRs. These toxins are found within venoms of different animals, such as snakes and *cone* snails, and some of them display a high affinity in the nanomolar range and/or selectivity towards nAChR subtypes. The structural determinants relevant to receptor binding and their potential for the development of highly potent and selective nAChR ligands are also presented and discussed.

In conclusion, in this Special Issue, the different aspects of the modulation of cholinergic signaling applied to several research fields are collected. I expect that, in the near future, the results presented herein will inspire further investigations in this research field towards the identification of new cholinergic ligands with additional therapeutic potential.
